# Commercial oats in gluten-free diet: A persistent risk for celiac patients

**DOI:** 10.3389/fnut.2022.986282

**Published:** 2022-10-06

**Authors:** Juan Manuel Rodríguez, Virginia Estévez, Karla Bascuñán, Jimena Ayala, Magdalena Araya

**Affiliations:** ^1^Institute of Nutrition and Food Technology (INTA), University of Chile, Santiago, Chile; ^2^Department of Nutrition, Faculty of Medicine, University of Chile, Santiago, Chile; ^3^Corporación de Apoyo al Celíaco (COACEL), Santiago, Chile

**Keywords:** oats, gluten-free, celiac disease, gluten-related disorders, gluten-free foods

## Abstract

**Background:**

Gluten-free diet is the treatment of celiac disease and other gluten-related disorders and excludes wheat, rye, and barley, while oats inclusion/exclusion has long been a matter of debate. A logo or catchphrase indicating the gluten-free condition in a product is all the consumer relies on to accept the product as suitable for his/her treatment. The oat-based gluten-free products represents a small market, which may have changed, and become more limited during COVID-19 pandemic.

**Objective:**

To assess gluten contamination in all labeled oat-based gluten-free local and imported products available in the market, comparing them to matched regular gluten containing counterparts. As a secondary objective, unconventional flours available in the same sale points were also assessed.

**Results:**

The search yielded 25 gluten-free labeled oat flours, rolled, and instant cereals, which were compared to 27 regular gluten containing equivalents. Gluten content was above the local (5 ppm) and the Codex Alimentarius cutoff (20 ppm) in 40 and 36% of the gluten-free labeled products, respectively. When all positive products were analyzed together, there were no differences in gluten content between labeled and unlabeled products. Locally produced products were more expensive, while rolled/instant oats were less contaminated than flours (*p* = 0.01). Precautionary labels advising presence of gluten as allergen was omitted in 37.0% of regular products. Only 33.3% of unconventional flours obtained from open markets and sold in bulk, were gluten contaminated.

**Conclusion:**

Oat-based gluten-free products are currently highly contaminated. It is urgent to regulate them and implement protocols that allow safe consumption of these products.

## Introduction

The gluten-free diet is the only treatment of gluten-related disorders (celiac disease, non-celiac wheat/gluten sensitivity, and wheat allergy) and requires exclusion of wheat, rye, and barley from the diet (1). Inclusion or exclusion of oats in a gluten-free diet has long been a matter of controversy (2). The equivalent storage proteins in barley, rye and oats differ in some aspects from wheat gluten (3). In avenin, in oats, contain shorter stretches of proline and asparagine (Pro + Asn) typical for gluten molecules. Avenins represent a protein family of at least 10 members and the evidence published indicate that some contain gluten-reactive epitopes. Independent of whether avenin can cause damage to celiac patients and should remain a concern (see “Discussion”), gluten contamination of oats-based products deserves attention because of the highly frequent contamination they suffer during processing and distribution. At present this is the main reason why commercial oats-based products remain being a concern among health professionals dealing with patients that require gluten-free diet as treatment. Oats have hypocholesterolemic properties, cardiovascular benefits through positive effects on blood glucose level, and help controlling body weight and blood pressure (4, 5). Also, oats consumption increases fecal mass, thus alleviating constipation; in addition, oats contribute to the maintenance of a balanced microbiome, attributed to its high content of soluble fiber (mainly beta-glucans specific to oats) (4–6). Due to these positive effects and the fact that gluten-free products are often low in vitamins, minerals and especially fibers, are high in starch and salt, and contain more saturated fat and many additives, inclusion of oats in gluten-free diet is often advised by many specialists (7).

To satisfy gluten-free diet followers needs, the food industry has developed a growing gluten-free foods market. To assure that these products remain safe for treatment, the Codex Alimentarius norms their characteristics (8); they must be free of wheat, rye and barley, and the gluten content in the final product must be below 20 mg/kg of product. In several countries though, the cut off is lower than this, as in Chile (9) and Australia (10), where this is 5 and 3 mg/kg of product, respectively. Guidelines and norms define the gluten content in the final product, but they do not refer to how to treat potential gluten contamination along production and how to avoid it. Indeed, although oats as substitute appears indeed nutritionally attractive, all benefits of including them in gluten-free diet are lost when gluten contamination occurs.

In Canada, in 2011, a study reported that the conventional commercial oat supply is heavily contaminated with gluten from other grains: 88% of regularly produced oat samples were contaminated above 20 ppm gluten (11). In the USA, 75% of gluten-free labeled oat samples were contaminated too (12). A recent study by our group in apparently healthy adult population in Santiago revealed that during the COVID-19 pandemic, patients and other consumers following gluten-free diet developed some strategies to cope with shortage of gluten-free foods, higher prices of those available, and diminished income due to quarantines (13). They decreased purchasing processed foods and favored cooking at home, which in turn increased the need of gluten-free cooking ingredients; among the most frequently mentioned were whole oats, together with naturally gluten-free flours from flaxseed, chickpeas, almond, coconut, rice, and nuts (data not published).

Taking into consideration all these data and considering that COVID-19 pandemic probably modified the market of gluten-free foods and deteriorated safety supervision programs, we set as objective to this study to assess gluten contamination in all labeled gluten-free oats flours and cereals containing only oats, presented as rolled and instant oats (locally produced and imported), available in the market. As a secondary objective, a small set of unconventional flours available in the same sale points were also assessed.

## Methods

During January–March 2021, shortage of food was noticeable in the city due to the COVID-19 pandemic. A thorough search of oat flours and oats presented as “rolled” and “instant” cereals was performed, including the online markets and shops offering them; data was collected in large, medium size and small supermarkets and health food stores. Other oats-based products (bars, breakfast cereals, and others) contained several additional ingredients and therefore were excluded. Front and back phases in the package were photographed. Name, origin, type of process (flour, rolled or instant oats), weight, brand, logo, or sentence describing the gluten-free condition, allergen declaration and price were registered. Gluten-free labeled products were matched with unlabeled, regular products that contain gluten/wheat/rye/barley (from now on referred to as “gluten containing products”), equivalent by type, brand, and weight. Gluten content was measured using a R5 sandwich ELISA test (Ridascreen^®^ Gliadin R7001). Results were analyzed by means of descriptive statistics, as needed.

## Results

The search resulted in 25 gluten-free labeled oats flours, rolled, and instant cereals, which were compared to 27 regular gluten containing equivalents (Table 1). Gluten content was above the local (5 ppm) and the Codex Alimentarius cutoff (20 ppm) in 40 and 36% of the gluten-free labeled products, respectively (Table 2). Among regular, unlabeled products only 66.7% contained gluten exceeding the Codex Alimentarius cutoff. National and imported (mainly from USA, Italy, Argentine, and Spain) products carried a variety of catchphrases or symbols informing their gluten-free condition; the analyses showed no association between type of labels and degree of contamination (NS). Taking all products (labeled and unlabeled) that were over 5 ppm gluten, the analysis showed no differences depending on the presence or absence of a “gluten-free” declaration, but again rolled/instant oats were less contaminated than flours (*p* = 0.025) (Supplementary Table 1). When the role of processing was assessed by comparing gluten-free flours, rolled and instant oats, no differences were detected either (*p* = 0.06), but comparison of flours against rolled plus instant oats showed that the latter were less likely to be contaminated (*p* = 0.02), the difference being mainly due to the absence of contamination among instant products (Table 3). Imported products were less contaminated than local ones (*p* = 0.01), with none of the instant imported oats being contaminated (Table 3). Gluten-free products are generally more expensive than gluten containing ones, therefore, we analyzed the role of price in gluten contamination. No differences were detected either by local/ imported or flour/rolled/instant condition.

**Table 1:** Gluten-free labeled and regular gluten containing products assessed, by local/imported origin, presence/absence of a gluten-free identification label, and allergen declaration.

**Product**	**N**	**National**	**Imported**		
**All products assessed**					
Total	52	42	10		
Oats flours	14	13	2		
Other oats	38	29	8		
Rolled	26	19	6		
Instant	12	10	2		
**Label information**				**Gluten-free/ precautionary declaration**	**Allergen declaration**
**Gluten-free labeled products**					
Total	25	18	7	25	0
Oats flours	8	7	1	8	0
Other oats	17	11	6	17	0
Rolled	13	9	4	13	0
Instant	4	2	2	4	1
**Regular products**					
Total	27	24	3	0	17
Oats flour	6	5	1	0	3
Other oats	21	18	2	1	15
Rolled	13	10	0	1	8
Instant Oats	8	8	1	0	7

**Table 2:** Percentage distribution of assessed products (by gluten content in ppm) in all products assessed, gluten-free labeled, and gluten containing products.

**Products**	** < 5 ppm**	**> 5 to < 20 ppm**	**> 20 ppm**
**All products assessed**			
Total	46.2	1.9	51.9
Oats flours	21.4	0	78.6
Other oats	55.3	2.6	42.1
Rolled	53.8	3.8	42.4
Instant Oats	58.3	0	41.7
**Gluten-free labeled products**			
Total	60	4	36
Flour	25	0	75
Other Oats	76.5	5.9	17.6
Rolled	69.2	7.7	23.1
Instant Oats	100	0	0
**Regular products**			
Total	33.3	0	66.7
Oats flour	20	0	80
Other oats	38.1	0	61.9
Rolled	38.5	0	61.5
Instant Oats	37.5	0	62.5

**Table 3:** Comparison of gluten-free labeled products negative or positive for gluten (in percentage), divided by local-imported, and above-under the mean category price.

**Products**	**Negative**	**Positive**	***p***	**Local**	**Imported**	***p***	**Above**	**Under**	***P***
**All**			0.06						
Total	60	40	0.44	55.1	10	**0.02**	20	46.7	0.1
Flour	25	75	0.79	84.6	0	0.25	66.7	100	0.17
Bulk Oats	76.5	23.5	**0.02**	55.2	22.2	0.24	12.5	33.3	0.31
Regular Oats	69.2	30.8	0.52	57.9	28.6	0.23	20	37.5	0.51
Instant Oats	100	0	–	50	0	–	0	0	–

Significant values are shown in bold.

Six unconventional flours (rice, flaxseed, chickpea, almond, coconut, and walnut) were found at the open markets included in the study, all were sold in bulk, which represents the worst-case scenario for gluten cross contact; only chickpeas and rice flours were contaminated (33.3%), their gluten content ranging between 22 and 39 ppm.

## Discussion

The two most relevant findings in this study are the high proportion of gluten-free labeled oats foods contaminated with gluten (40 and 36% depending on the cutoff used), and that when positive gluten/free and gluten containing products, labeled and unlabeled were compared, no differences were detected in the magnitude of gluten content (NS).

Publications on gluten contamination of oats are scarce in recent years. In Spain (2006) and USA (2011), results obtained revealed that approximately 75% of labeled products were contaminated, while in Canada and India, 88% and 85% of products proved to have high gluten levels (11, 12, 14, 15). To produce safe oats appropriate for gluten-free diets, Canada and the USA developed a “purity protocol” (which includes good manufacturing practices and Hazard Analysis of Critical Control Points), which aims at warranting avoidance of gluten contamination during cultivars, harvest, storage, processing, handling, and transportation (16). This protocol usually includes testing gluten content along the production processes and in the final product, to verify it remains below the accepted limits. Also, this protocol is auditable for gluten-free certification and leads to an accurate labeling. Yet, it is not applied everywhere and in the present study, lack of application of this protocol may explain at least in part that locally produced gluten-free oats were more contaminated than imported ones. This in turn reflects the lack of priority and good public policies to survey and certify gluten-free products that are still present here and in many other countries. Gluten presence in gluten-free labeled oats is not only mislabeling but also against regulations and put at risk patients for whom gluten avoidance is the only efficacious treatment. Locally produced products were also more expensive than imported ones, which confirms the well-known higher costs that celiac patients must afford to maintain a strict gluten-free diet (17–21). Improving domestic production is something that clearly must be promoted, and celiac patients must learn how to choose labels and brands to ensure their diet remains safe.

Gluten containing cereals based on wheat, barley, and rye are among the eight most common food allergens identified by the Codex Alimentarius and many food country regulations. It is relevant then that independent of the fact that declaring gluten presence as ingredient is mandatory, 26% of the regular products did not mention the presence of allergens, omission that also mislead allergic patients to consume unsafe products. It was unexpected that only 33.3% of the unconventional flours, which were obtained from open markets and sold in bulk, were gluten contaminated (22 ppm and 39 ppm of gluten in chickpea or rice flour, respectively), but results of this study do not clarify at which point contamination occurs. That almond flour showed no contamination (<5 ppm) may be influenced by the fact that it is usually processed in mills dedicated to oily matrices, because its rich fat components are suboptimal to share mills with cereal flours.

The main limitation of this study is the restricted number of samples obtained. However, all products available in the market were included, so this rather may be interpreted as a relevant result reflecting the effects of the confinement period due to COVID-19 pandemic, not only on shortage of oats gluten-free food, but on food shortage in general. In Mexico, other authors have also described a decrease of imported products during the pandemic period (22).

Another issue interesting to discuss is that dealing with gluten-free oats is not simple. Contamination can occur at different stages, a relevant one being the rotation of cereal crops (wheat, barley, rye) with legumes such as chickpeas, lentils, or beans; this is a common practice because the latter help fixing nitrogen in the soil (22). Moreover, they are harvested with the same equipment, stored, processed, transported, and distributed together (23). Allred et al. defined that packaging also plays a role, considering that today many small producers use fragile materials for packaging. In the case of regular oats, contamination of 1% with other grains is generally allowed, but must be controlled.

Today, the possibility of safe oats production has been widely discussed (“purity protocol”) and it is generally accepted that it is feasible. The requirement is that oats are planted on ground that did not have gluten containing cereals for the last 3 years. Near harvest time, the grower walks through the fields and pulls out any stray of gluten grains. This is possible because of the short stature of this proprietary oat variety. Wheat, rye and barley are taller and easy to see. Later, an inspector walks around the field, certifying that is clean. Harvesting is done with certified gluten-free associations that only deal with gluten-free oat production. These oats should be stored in new bags or certified clean bins to avoid other sources of cross contamination, and carefully processed in exclusive equipment's and gluten-free facilities. The successful application of the “Purity Protocol” has shown that is indeed possible to produce safe oats in compliance with the legislation and suitable for celiac patients on gluten-free diet (23). Thus, although there is robust evidence indicating that oats are indeed frequently contaminated (15, 22, 24), at the same time improving the practice of using special gluten-free oats cultivars and supervision of oat production and manufacturing appears as a potent line of action to obtain good, safe products.

It is also interesting to discuss that one thing is to have uncontaminated gluten-free oats and another that the oats avenin proper be safe for all celiac patients. In fact, whether oats avenins can elicit the inflammatory processes characteristic of CD is still uncertain, and the biological consequences of oats consumption by all celiac patients remains controversial. In 1995 Janatuinen reported that celiac patients in remission consuming 49.9 ±14.7 g per day for 6 months and newly diagnosed patients consuming 46.6 ± 13.3 g per day for 12 months did not differ in nutritional status, symptoms, or laboratory measures when compared to controls (25). These kind of data and others obtained in subsequent years supported the idea that oat consumption is safe for celiac patients. However, different oats cultivars have demonstrated to be able to initiate the inflammatory process in controlled experimental conditions. This has been measured in K562(S) cells, by electrical resistance of T84-cell monolayers and other techniques (26). A study conducted in small groups of celiac patients showed that some celiac patients (27) have avenin-reactive mucosal T-cells that can cause mucosal inflammation (27). So, unless the specific oat cultivars used in specific food products are declared, is not possible to assure oats' safety for all patients.

A last topic that deserves attention is the methodology used to measure gluten contamination. In this study we used the AOAC-approved quantification methodology, and with this technique when gluten content in the sample exceeds the kit limit, it cannot be further quantified and therefore determining the magnitude of gluten contamination is limited. Neither is clear the participation of rye and barley gluten equivalent molecules with the currently used kits. So, putting together all aspects reviewed, and until life conditions imposed by the COVID-19 pandemics return to normal, it seems reasonable to advice patients on gluten-free diet to be cautious when deciding whether to include oats in their diet. It is difficult to solve the problems unless manufacturers and health authorities are involved. The food industry must commit to use certified gluten-free raw materials and following Good Manufacturing Practices and Hazard Analysis of Critical Control Points, while health authorities should effectively control gluten-free oats-based food production.

## Data availability statement

The original contributions presented in the study are included in the article/Supplementary materials, further inquiries can be directed to the corresponding author.

## Author contributions

Conceived and designed the study: JR, VE, JA, KB, and MA. Results analyses: JR, VE, and MA. Intellectual contribution and preparation of drafts: JR, VE, KB, and MA. MA wrote the final manuscript. All authors contributed to the article and approved the submitted version.

## Conflict of interest

The authors declare that the research was conducted in the absence of any commercial or financial relationships that could be construed as a potential conflict of interest.

## Publisher's note

All claims expressed in this article are solely those of the authors and do not necessarily represent those of their affiliated organizations, or those of the publisher, the editors and the reviewers. Any product that may be evaluated in this article, or claim that may be made by its manufacturer, is not guaranteed or endorsed by the publisher.
