# Supplementary material for: Commercial oats in gluten-free diet: A persistent risk for celiac patients
Source: Front Nutr. 2022 Oct 6;9:986282. doi: 10.3389/fnut.2022.986282 (PMC9582257; doi:10.3389/fnut.2022.986282)
Supplement: Supplementary file 1 [file Table_1.docx]

| **Supplementary Table 1.** Comparison (in percentage) of gluten positivity in gluten-free labeled or regular ones. | | | | | | | | |
| --- | --- | --- | --- | --- | --- | --- | --- | --- |
| **Products** | | **Gluten-free labeled** | | **Regular products** | | **Student’s t test** | |  |
| **All** | |  | |  | |  | |  |
| Total | | 40 | | 66,7 | | 0,09 | |  |
| Flour | | 16,7 | | 83,3 | | 1 | |  |
| Bulk Oats | | 23,5 | | 61,9 | | **0,025** | |  |
| Regular Oats | | 30,8 | | 61,5 | | 0,23 | |  |
| Instant Oats | | 0 | | 62,5 | | - | |  |
|  |  | |  | |  | |  |  |

Significant values appear in bold
